# Episodes of violence suffered by migrants transiting through Libya: a cross-sectional study in “Médecins du Monde’s” reception and healthcare centre in Seine-Saint-Denis, France

**DOI:** 10.1186/s13031-020-0256-3

**Published:** 2020-02-28

**Authors:** L. Reques, E. Aranda-Fernandez, C. Rolland, A. Grippon, N. Fallet, C. Reboul, N. Godard, N. Luhmann

**Affiliations:** Médecins du Monde, 62 rue Marcadet, 75018 Paris, France

**Keywords:** Migration, Violence, Libya, Healthcare access

## Abstract

**Introduction:**

The Central Mediterranean Route, passing through Libya, is one of the most dangerous for migrants. Episodes of violence have been documented but have not been accurately quantified. The objective of the study was to estimate the prevalence of episodes of violence suffered in Libya by migrants consulting the Médecins du Monde reception and healthcare centre in Seine-Saint-Denis (Ile-de-France).

**Methodology:**

A monocentric cross-sectional study was conducted from February to May 2019 including migrants over the age of 18 years who had passed through Libya and arrived in Europe from 2017. The presence of emotional distress was considered as exclusion criterion. The proportion, frequency and factors associated to physical, deprivation and sexual violence in Libya were estimated through a bespoke questionnaire, as well as healthcare access in Libya and psychosocial support needs.

**Results:**

Ninety eight people were recruited and 72 were interviewed (17 refused to participate and 9 were excluded). 76.4% were men, with a mean age of 31.9 years, 76.4% had low educational level, 66.7% came from Ivory Coast and 59.7% had left their country for security reasons. The median length of stay in Libya was 180 days. The overall proportion of participants having suffered from violence was 96.4% among men and 88.2% among women. The prevalence of physical, deprivation and sexual violence for men and women were 94.2, 81.7 and 18% and 80.0, 86.7 and 53.3%, respectively. Access to healthcare in Libya was 2.8 and 63.9% of participants were oriented to psychosocial support after the interview.

**Conclusions:**

The vast majority of migrants reported having been victims of violence during their transit through Libya. Women were at particular risk of sexual violence. Access to health care in Libya was almost non-existent. Psychosocial support for this population is urgent.

## Background

Several reports show that violence related to migration, including physical assault, torture and sexual violence, is a sad reality [1–3]. Among the migratory routes, the Central Mediterranean, which crosses Libya, is one of the most dangerous [2] .

Libya has been frequently indexed by the media for its dysfunctional migration policy and several international organisations have collected evidence and testimonies recounting the violence suffered by migrants [4–6]. Nevertheless, there are no scientific studies collecting quantitative evidence on this subject.

The objectives of this study were to quantify the violence episodes according to their typology; to determine its associated factors, as well as to assess healthcare access and need for psychosocial support among migrants having transited Libya and visiting the Médecins du Monde (MdM) reception and healthcare centre in Seine-Saint-Denis (Ile-de-France).

## Methods

The study was cross-sectional and monocentric. The target population was the beneficiaries of the MdM reception and healthcare centre in Seine-Saint-Denis. Individuals aged over 18 years old, with foreign nationality, having transited through Libya and having arrived in Europe from 2017 were included. Participants were excluded if they presented emotional distress, due to the risk of further psychological distress linked to the evocation of traumatic events. The presence of emotional distress was assessed by the Refugee Health Screener-15 (RHS-15) [7].

Patients were recruited during medical consultations. Once they had consented to participate they were interviewed in a confidential space by an specialised doctor, who had previously evaluated the exclusion criteria.

A sample size of 72 individuals was set according to a prevalence of violence of 95%, a margin of error of 5% and a confidence interval (CI) of 95%. Sampling was exhaustive until accomplishing the sample size.

The data collection tool was a bespoke online survey (KoboTool Box). Data were automatically added to a secure database. A two-weeks pre-test period was conducted in order to test the survey feasibility.

The study was conducted in French and assessed for two types of violence: direct and witnessed. In both cases, physical, sexual and deprivation violence episodes were tracked. Physical violence was divided in “episodes with firearms” (i.e. injury, aggression, shooting) and “episodes without firearms” (physical injury, beatings, threat). Sexual violence addressed “episodes of rape”, deprivation violence included suffering from or witnessing “episodes of detention”, “episodes of food deprivation”, “episodes of confiscation”, “episodes of racketeering”, and “episodes of separation of family members”. The frequency and the perpetrators of these episodes were also tracked. For the former it was set to “daily”, “weekly”, “less than once a week” or “very rare” and the latter was divided in “governmental forces”, “armed groups”, “civils” or “not identified”. Healthcare access during the journey in Libya and need for psychosocial support after the interview were also assessed. Participants were oriented to psychosocial specialised teams after their agreement.

The descriptive analysis for continuous variables used mean ± standard deviation, median, interquartile range and Student T tests. Percentages with a 95% confidence interval and Chi2 tests were used for categorical variables. Stata v.15 software was used for statistical analysis.

This study was conducted in compliance with the ethical principles of the Helsinki Declaration. All participants received a written information sheet and their participation was voluntary after providing verbal informed consent. All information provided was anonymised and confidentiality was ensured. Participants could stop and/or refuse to continue the survey at any moment. Psychosocial support was available throughout the interview.

### Main findings

Ninety eight people were recruited and 72 were interviewed from February to May 2019 (17 refused to participate and 9 were excluded due to emotional distress).

Table 1 describes the population and the episodes for the different violence types. Seventy two people were interviewed (55 men and 17 women). The mean age was 31.9 years old, 76.4% had primary studies, 66.6% came from Ivory Coast (followed by Mali 23.6%) and 29.4% of women were pregnant at some stage of the journey. The mean length of stay in Libya was 180 days and only 2.8% had access to healthcare during the stay. Sixty three point 9 % of the participants looked for psychosocial support after the interview. Statistically significant differences by sex were found for the educational level (*p* = 0.03), the type of travel (*p* = 0.01) and healthcare access (*p* = 0.05).

**Table 1:** Sociodemographic characteristics and episodes of different types of violence in Libya by sex

	Total (*N* = 72)		Women (*N* = 17)		Men (*N* = 55)		*p* value
SOCIODEMOGRAPHIC CHARACTERISTICS							
Age in years (mean, SD)	31,9	(7,4)	30,2	(7,8)	32,5	(7,3)	0,27
Educational level (%, n/N)							
Primary	76,4	(55/72)	100,0	(17/17)	69,1	(38/55)	0,03*
Secondary	18,1	(13/72)	0,0	(0/17)	23,6	(13/55)	
Tertiary	5,5	(4/72)	0,0	(0/17)	7,3	(4/55)	
Country of Origin (%, n/N)							
Ivory Coast	66,6	(48/72)	82,4	(14/17)	61,8	(34/55)	0,51
Mali	23,6	(17/72)	17,6	(3/17)	25,5	(14/55)	
Eritrea	1,4	(1/72)	0,0	(0/17)	1,8	(1/55)	
Republic of Cameroon	5,6	(4/72)	0,0	(0/17)	7,3	(4/55)	
Reasons for leaving country of origin (%, n/N)							
Security	59,7	(43/72)	82,3	(14/17)	52,7	(29/55)	0,12
Political	15,3	(11/72)	5,9	(1/17)	18,2	(10/55)	
Economic	22,2	(6/72)	11,8	(4/17)	25,5	(14/55)	
Studies	2,8	(2/72)	0,0	(0/17)	3,6	(2/55)	
Type of travel (%, n/N)							
Alone	69,4	(50/72)	41,2	(7/17)	78,2	(43/55)	0,01*
With family	19,4	(14/72)	35,3	(6/17)	14,5	(4/55)	
Other	11,1	(8/72)	23,5	(4/17)	7,3	(8/55)	
Pregnancy during travel (%, n/N)	_	_	29,4	(5/17)	_	_	_
Boarding by threat (%, n/N)	58,1	(42/72)	47,1	(8/17)	61,8	(34/55)	0,68
Length of stay in Libya in days (median, IQR)	180	(120–365)	120,0	(60–180)	200	(120–365)	0,02*
Healthcare access in Libya (%, n/N)	2,8	(2/72)	0	(0/17)	3,6	(2/55)	0,05*
Need of psychological support after the survey (%, n/N)	63,9	(46/72)	70,8	(12/17)	61,8	(34/55)	0,35
EPISODES OF DIRECT VIOLENCE (%, CI 95%)							
Global Violence^1^	94,4	(89,0-99,9)	88,2	(71,3–100)	96,3	(91,3–100)	0,21
Physical violence	91,1	(84,3-98,1)	80,0	(57,1–100)	94,3	(87,9–100)	0,05*
With firearms	76,1	(65,6-86,6)	53,5	(24,7-81,9)	82,7	(72,1-93,3)	0,02*
Without firearms	89,5	(82,1-97,1)	73,3	(48,0-98,7)	94,3	(87,9–100)	0,02*
Violence with deprivation	89,5	(82,1-97,1)	86,7	(67,7–100)	91,7	(85,6–100)	0,10
Detention without food	88,2	(79,3-98,1)	86,7	(67,7–100)	88,7	(78,3-99,0)	0,85
Detention with racket	58,8	(46,1-71,5)	46,7	(18,1-75,2)	62,3	(47,7-76,8)	0,31
Detention with ransom	58,8	(46,1-71,5)	60,1	(31,9-88,0)	58,5	(43,8-73,2)	0,92
Family separation	37,6	(24,2-49,2)	46,7	(18,1-75,2)	34,0	(19,7-48,2)	0,40
Sexual violence	26,5	(15,7-37,2)	53,3	(24,7-81,9)	18,9	(7,9-24,8)	0,01*
Other types of violence							
Forced labour	33,3	(19,9-47,5)	22,2	(9,8-35,6)	49,1	(17,5-82,1)	0,03*
Unsafe abortion	_	_	17,6	(5,3-29,9)	_	_	_
EPISODES OF WITNESSED VIOLENCE (%, CI 95%)							
Global Violence^1^	100,0	(100–100)	00,0	(100–100)	100,0	(100–100)	_
Physical violence	93,1	(85,9–100)	88,2	(71,1–100)	94,3	(87,9–100)	0,46
With firearms	87,5	(78,7-93,2)	82,4	(62,1–100)	89,1	(79,1-99,0)	0,52
Without firearms	93,1	(85,9–100)	88,2	(71,1–100)	92,7	(83,9–100)	0,87
Violence with deprivation	94,4	(89,0-99,9)	88,2	(71,3–100)	96,3	(91,3–100)	0,21
Detention without food	87,5	(78,3-96,5)	88,2	(71,2–100)	87,3	(76,8-97,7)	0,93
Detention with racket	76,4	(65,6-87,1)	76,4	(54,0-99,0)	76,4	(63,7-89,1)	0,99
Detention with ransom	79,2	(68,8-89,6)	70,6	(46,4-94,7)	81,8	(70,1-93,6)	0,37
Family separation	61,1	(48,9-73,3)	52,9	(26,5-79,4)	63,6	(49,5-77,6)	0,46
Sexual violence	79,2	(68,8-89,6)	82,4	(62,1–100)	78,2	(65,8-90,6)	0,74
Other types of violence							
Forced labour	88,2	(79,3-98,1)	86,7	(67,7–100)	88,7	(78,3-99,0)	0,85

1 The term Global violence refers to physical and/or deprivation and/or sexual violence *p values correspond to two sample T-tests for continuous variables and to Chi-2 tests for categorical values. Values with * are statistically significant (*p* < 0.05). *SD* Standard deviation, *IQR* Interquartile range

According to the episodes of direct and witnessed violence, results show that 94.4% of the participants suffered some type of violence (93.6% of men and 88.2% of women). Ninety one point 1 % of participants suffered from physical violence (80.0% of women and 94.3% of men). Eighty nine point 5 % of participants experienced violence with deprivation (81.7% of men and 86.7% of women) and 26.5% suffered sexual violence (18.9% of men and 53.3% of women). In addition, 49.1% of men and 22.2% of women experienced forced labour and 17.6% of women had unsafe abortions during the journey. Differences by sex were statistically significant for physical, sexual violence and forced labour (*p* < 0.05).

One hundred percent of participants witnessed some kind of violence, with 93.1% witnessing episodes of physical violence, 94.4% episodes of deprivation violence, 79.2% episodes of sexual violence and 88.2% other type of episodes of violence. No statistically significant differences by sex were found.

Table 2 describes episodes of physical, deprivation and sexual violence by sociodemographic characteristics and other secondary variables. Apart from differences by sex, no statistically significant differences were found by other variables.

**Table 2:** Episodes of direct physical, deprivation and sexual violence by secondary variables

	Global violence^1^			Physical violence			Deprivation violence			Sexual violence		
(N = 72)	No	Yes	*p*	No	Yes	*p*	No	Yes	*p*	No	Yes	*p*
Sex (%)												
Male	3,4	96,4	0,20	5,7	94	0,08	15,1	84,9	0,87	81,1	18,9	0,01*
Female	11,6	88,2		20	80		13,3	86,7		46,7	53,3	
Age in years (mean, SD)	30	32	0,53	31	31,8	0,75	32	30,5	0,61	30,7	34,4	0,71
	(5,0)	(7,6)		(7,0)	(8,6)		(6, 7)	(7,9)		(6,1)	(8,6)	
Level of Education (%)												
Primary	5,5	94,6	0,16	7,7	92,3	0,31	11,5	86,5	0,82	71,2	28,9	0,52
Secondary	0	100		7,7	92,3		15,4	84,6		76,9	23,1	
Tertiary	25	75,0		33,3	66,7		33,3	66,7		100,0	0	
Country of Origin (%)												
Ivory Coast	4,1	95,8	0,09	8,7	90,3	0,8	13,0	84,8	0,98	80,4	19,6	0,17
Mali	5,9	94,1		6,5	93,8		18,8	81,3		50,0	50	
Eritrea	0	100		25	75		0,0	100		100,0	0	
Republic of Cameroon	33,3	66,7		0	100		0,0	100		75,0	25	
Type of travel (%)												
Alone	6	94	0,45	6,4	93,6	0,57	14,9	83	0,79	74,5	25,5	0,55
With family	0	100		14,3	85,7		14,3	85,7		64,5	35,7	
Other	12,5	87,5		14,3	85,7		0,0	100		85,7	14,3	
Length of stay in Libya (mean, SD)	118,3	259,5	0,79	122,5	270,3	0,81	104,3	277,4	0,83	109,3	268,5	0,86
	(70,1)	(116,2)		(72,1)	(126,6)		(76,1)	(135,9,2)		(65,1)	(126,2)	

*p values correspond to two sample T-tests for continuous variables and to Chi-2 tests for categorical values. Values with * are statistically significant (*p* < 0.05). *SD* Standard deviation

In addition, related to the frequency of physical violence episodes, 38% of participants reported daily violence, 48% weekly violence, 15% less than weekly and 5% rarely. These percentages were 37, 54, 9 and 0% for deprivation violence and 41, 25, 34 and 0% for sexual violence, respectively. In 54% of the cases perpetrators were not identified, 29% were identified as civils, 10% as armed groups and 7% as governmental forces.

## Discussion

Human rights’ violations in Libya have been documented for years by international organizations, think tanks and journalists [4, 8–10]. Nevertheless, this evidence come mainly from individual testimonies, reports or press releases. The added value of our study resides in its methodology which enables the quantification of the prevalence of these episodes in migrants hosted in a European country when arriving to a reception centre.

Our results show that the vast majority of people suffered from long imprisonment and confinement, where violence was systematic and of a huge magnitude. In most cases, the episodes of physical violence were suffered every day or almost every day and deprivation episodes lasted for the whole journey. In addition, perpetrators were very difficult to identify (more than a half of the participants simply called them “the Arabs”).

Several NGOs and international agencies have reported that physical violence occurs mainly in places of confinement and is closely linked to the exploitation of migrants, forced labour and extortion [4, 11, 12]. Firearms are very common and often used for threat, but episodes of mass murders and shootings have also been documented. Several reports mention extortion as a common practice. However, NGOs note that migrants are often abducted by smugglers when they arrive in the country. They can be released if they pay, themselves or their families but they can also be sold to another smuggling group [4, 11].

In our study, women were particularly vulnerable to sexual violence. This has also been documented by other reports pointing that sexual violence is a common practice during the detentions or before being released [13–16]. One third of the victims of sexual violence of the study stated that episodes were very common (daily or almost daily and by multiple perpetrators). Nevertheless, this type of violence might have been underestimated, especially for men, as the nature of the violence, which affects their privacy, sexuality and gender identity, makes it difficult for them to speak up.

Our study shows that none of the participants had ever been treated by a doctor or had received medication during their journey. It has been documented that the health system in Libya has collapsed and it is facing serious problems due to infrastructure damage, lack of medicines, medical equipment and staff [10, 17]. 

Most people asked for psychological support after the questionnaire, reflecting a real urgency. The literature has well documented how mental health disorders occur as a consequence of migration, mainly linked to forced, unplanned, poorly planned or illegal migration, low educational level, isolation, lack of support and perceived discrimination [18–20].

### Limitations

The profile of participants is restricted to the recruitment location, which limits the external validity of the study. In addition, it is reasonable to assume that participants with emotional distress may have experienced higher levels of exposure to violent events and therefore their exclusion from the study may have resulted in an under-estimate of overall levels of violence exposure. Sexual violence might also have been underestimated, especially among men, as it was normally evoked as witnessed. Finally, the low proportion of women could limit the statistical power for this group.

## Conclusions

Our study of migrants presenting at the MdM reception and healthcare centre in Seine-Saint-Denis showed that the vast majority were victims of violence during their transit through Libya. Women were at particular risk of sexual violence. Access to health care in Libya was almost non-existent. Psychosocial support for this population is urgent.

## Data Availability

The datasets used and/or analysed during the current study are available from the corresponding author on reasonable request.

## Abbreviations

CI: Confidence Intervalle
INSERM: Institut national de la santé et de la recherche médicale
IQR: Inter Quartile Range
IRB: Institutional Review Board
MdM: Médecins du Monde
NGO: Non-Governmental Organization
RHS-15: Refugee Health Screener 15
SD: Standard Deviation
